# The effectiveness of a multisite, multidisciplinary analgesic stewardship program: a 12-month retrospective cohort study

**DOI:** 10.1097/PR9.0000000000001339

**Published:** 2025-10-07

**Authors:** Jeremy Szmerling, Sam Maleki, Gordon Mar, Galahad Gu, Emily Schembri, Paul Buntine, Anne Goulopoulos, Patrick J. Owen

**Affiliations:** a Eastern Health Pharmacy Department, Melbourne, Victoria, Australia; b Eastern Health Acute Pain Service, Melbourne, Victoria, Australia; c Eastern Health Clinical School, Monash University, Melbourne, Victoria, Australia; d Eastern Health Emergency Medicine Program, Melbourne, Victoria, Australia

**Keywords:** Analgesics, Opioid, Pain management, Acute pain, Narcotics, Drug utilization, Medication therapy management, Patient safety

## Abstract

**Introduction:**

Safe and judicious use of opioids is essential for effective pain management and reducing associated harm.

**Objectives:**

We examined the effectiveness of a multisite, multidisciplinary analgesic stewardship program comprising of both organisation- and patient-level interventions (intervention) compared with organisation-level interventions alone (control).

**Methods:**

This retrospective cohort study included adult inpatients who were either opioid-naïve at preadmission and prescribed a modified-release opioid (opioid-naïve cohort; n = 183) or nonopioid-naïve at preadmission (nonopioid-naïve cohort; n = 577). The primary outcomes were discharge on a modified-release opioid (opioid-naïve cohort), change in oral morphine equivalent daily dose from preadmission to discharge (nonopioid-naïve cohort), and readmission within 2 weeks (both cohorts). Data were collected at preadmission and discharge over 12 months (August 2022 to July 2023).

**Results:**

Patients (n = 760, female: 63%) had a mean (SD) age of 71.9 (19.1) years and a hospital length of stay of 11.0 (14.6) days. Among the opioid-naïve cohort, the intervention led to a between-group reduction in discharge on modified-release opioids (OR [95% CI]: 0.23 [0.11–0.49], *P* < 0.001), yet not readmission within 2 weeks (OR [95% CI]: 0.76 [0.08–7.45], *P* = 0.813). Among the nonopioid-naïve cohort, the intervention led to a between-group reduction in readmission within 2 weeks (OR [95% CI]: 0.39 [0.15–0.98], *P* = 0.045), yet not oral morphine equivalent daily dose (estimated marginal mean net difference [95% CI]: −7.46 [−16.74 to 1.82] mg, *P* = 0.115).

**Conclusion:**

A multisite, multidisciplinary analgesic stewardship program comprising both organisation- and patient-level interventions may lead to better opioid prescribing at discharge and potentially clinically meaningful reductions in readmission when compared with organisation-level interventions alone.

## Abstract 2

Supplemental Digital Content is Available in the Text.

A multisite, multidisciplinary analgesic stewardship program may lead to better opioid prescribing at discharge and potentially clinically meaningful reductions in readmission.

## 1. Introduction

Opioids are commonly prescribed for pain management and global consumption continues to rise in high-income countries. ^15^ For example, opioids are often prescribed to patients with iatrogenic pain associated with surgical procedures. ^23^ However, 42% to 71% of dispensed tablets after surgery are neither used as intended nor disposed, ^7^ which subsequently creates a reservoir of opioids prone to nonintended use and questions the necessity of prescription. One Australian study showed that 70% of 580 patients prescribed opioids after surgery did not use all dispensed tablets and only 5% disposed the remaining tablets after cessation. ^1^ This is further compounded by the prescription of modified-release opioids that, while temporally on the decline in Australia, ^6^ offer limited benefit and heightened risk of adverse events compared with immediate-release opioids. ^17^ Therefore, identifying strategies to facilitate appropriate prescription of opioids is critical.

Analgesic stewardship programs aim to promote safe and effective use of opioids. ^20^ Evidence from a rapid review of 19 studies showed that health service-embedded analgesic stewardship programs may reduce opioid prescribing and associated adverse events. ^22^ We previously evaluated a single-site, 2-month trial (patients: n = 100) of a newly established multidisciplinary analgesic stewardship program implemented at a health service within metropolitan Melbourne, Australia. ^21^ Specifically, this study showed that among appropriately selected high-risk patients, the analgesic stewardship program led to cessation of modified-release opioids among approximately 90% (n = 23/26) of patients who were opioid-naïve at preadmission. ^21^ Our analgesic stewardship program also led to a mean (95% CI) reduction in oral morphine equivalent daily dose of 19.85 (−29.44 to −10.25) mg among patients who were nonopioid-naïve at preadmission. ^21^ Based on these findings, the analgesic stewardship program was funded beyond the initial 2-month trial phase and expanded to 3 further hospitals within the health service.

This study aimed to determine the effectiveness of a multisite, multidisciplinary analgesic stewardship program comprising both organisation- and patient-level interventions compared with organisation-level interventions alone on outcomes of opioid prescription (ie, discharge on a modified-release opioid, change in oral morphine equivalent daily dose from preadmission to discharge, and readmission within 2 weeks). Secondary aims examined whether site (hospital), unit (general medicine, surgical, or rehabilitation), or having a psychological comorbidity were associated with effectiveness.

## 2. Methods

### 2.1. Study design and setting

This 12-month retrospective cohort study was registered with the Eastern Health Human Research Ethics Committee (ID: QA23-127-104808) and reported following the STROBE statement (see Supplement A, supplemental digital content, http://links.lww.com/PR9/A348). ^10^ As per study conduct in line with the National Statement on Ethical Conduct in Human Research (Sections 2.3.9, 2.3.10), ^18^ a waiver of consent was approved for collection and use of retrospective deidentified patient data. Data were collected from patients discharged from 01 August 2022, to 30 July 2023, across 4 metropolitan hospitals (Box Hill Hospital: 61,674 admissions per year; Maroondah Hospital: 34,450 admissions per year; Angliss Hospital: 22,807 admissions per year; Peter James Centre: 12,387 admissions per year) within a single health service in Melbourne, Australia. According to the Australian Bureau of Statistics *Index of Relative Socioeconomic Advantage and Disadvantage*,^3^ these hospitals all represent socioeconomically advantaged geographical locations (seventh decile or greater).

### 2.2. Participants

Inclusion criteria (for both the intervention and control) were adult (aged 18 years and older) patients admitted to one of the included hospitals as an inpatient under a surgical, general medicine, or rehabilitation medical unit who were either: (1) opioid-naïve (not documented as being prescribed regular opioids [a drug that act on μ-opioid receptors in the central nervous system to provide analgesia, sedation, or euphoria, excluding peripherally acting agents such as loperamide]) at the point of admission and prescribed modified-release opioids (long-acting tablet, capsule, and transdermal patch formulations that release the drug gradually over an extended period) during their admission (*opioid-naïve* cohort) or (2) nonopioid-naïve (documented as being prescribed regular opioids) at the point of admission (*nonopioid-naïve* cohort). Exclusion criteria were patients who received clinical input/recommendation on analgesia from a specialist treating team (eg, acute pain service, palliative care, cancer services, or addiction medicine) or were prescribed methadone (methadone was excluded, as oral morphine equivalent daily dose cannot be accurately converted). Notably, these criteria contained additional restrictions to those in place for the existing hospital-embedded analgesic stewardship program. For the analgesic stewardship program, patients were identified through direct clinician referrals or a daily (weekdays, excluding public holidays) electronic medication management system report. The criteria for clinician referral included prescribed modified-release opioids, had acute or acute-on-chronic pain that is difficult to control, required complex discharge planning, would benefit from postdischarge follow-up regarding analgesia, or there were concerns regarding analgesia use.

### 2.3. Variables

Outcomes were discharge on a modified-release opioid (opioid-naïve cohort), change in oral morphine equivalent daily dose from preadmission to discharge (nonopioid-naïve cohort), and readmission within 2 weeks (both cohorts). Patients were exposed to either organisation-level analgesic stewardship program interventions only (*control* group) or organisation- and patient-level analgesic stewardship program interventions (*intervention* group).

#### 2.3.1. Control (organisation-level analgesic stewardship program interventions only)

Patients in the control group received organisation-level analgesic stewardship program interventions only. Organisation-level interventions included: establishment of the Analgesic Stewardship Service Sub-Committee that oversaw governance, policy development, and adherence to clinical guidelines; integration of stewardship protocols into the hospital accredited process based on compliance with the Australian Commission on Safety and Quality in Healthcare standards; integration of quality improvement initiatives into the electronic medical record system to facilitate analgesic management through standardised order sentences and duration setting for opioids; development of education resources that provided clinicians with access to analgesic stewardship training; benchmarking analgesic practices with external health services; and monthly key performance indicator monitoring using a scorecard that reports metrics to the Medication Management Clinical Governance Committee focusing on opioid prescribing practices, multimodal analgesic use, and discharge communication quality.

#### 2.3.2. Intervention (organisation- and patient-level analgesic stewardship program interventions)

Patients in the intervention group received both organisation- and patient-level analgesic stewardship program interventions. Patient-level interventions included an initial pharmacist assessment, analgesia medication reconciliation, review of real-time prescription monitoring system (SafeScript), ^9^ and development of analgesia management plans. Complex patient cases were discussed at weekly meetings with senior specialists in anaesthesia, addiction medicine, and psychiatry.

### 2.4. Data sources and measurement

Data were routinely collected variables extracted through electronic medical records using REDCap electronic data capture tools hosted at Eastern Health. ^13,14^ Data were collected at preadmission and discharge.

#### 2.4.1. Discharge on a modified-release opioid

Discharge on modified-release opioid (yes or no) was determined through chart audit.

#### 2.4.2. Oral morphine equivalent daily dose

Oral morphine equivalent daily dose (mg) was calculated using the Faculty of Pain Medicine, Australian and New Zealand College of Anaesthetists ^11^ opioid equianalgesic calculator.

#### 2.4.3. Readmission within two weeks

Readmission within 2 weeks (yes or no) was determined through chart audit.

#### 2.4.4. Descriptive data

Descriptive data were collected through chart audit and included: age (years), sex (male or female), smoking history (yes or no), alcohol use history (yes or no), illicit substance use history (yes or no), any psychological comorbidity (yes or no; per the DSM-5-TR ^2^), hospital length of stay (days), site (Box Hill Hospital, Maroondah Hospital, Angliss Hospital, or Peter James Centre), and unit (surgical, general medicine, or rehabilitation).

### 2.5. Study size

No a priori sample size calculation was performed. However, the study size was that of convenience over the 12-month study period whereby all eligible patients who received the intervention and a random sample of eligible patients who did not receive the intervention were included.

### 2.6. Quantitative variables

Site was collapsed to a binary outcome (Box Hill Hospital or non-Box Hill Hospital sites) due to comparative data paucity at non-Box Hill Hospital sites.

### 2.7. Statistical methods

All analyses were conducted in Stata (v17, StataCorp, College Station, Texas, United States of America). Patient characteristics were compared between cohorts (opioid-naïve and nonopioid-naïve) through independent *t* test, one-way analysis of variance, or χ^2^ test. Among the opioid-naïve cohort, odds of discharge on a modified-release opioid based on exposure to intervention or control was examined using univariate logistic regression. Among the nonopioid-naïve cohort, separate linear mixed models with random effects (patients) examined within- and between-group (intervention and control) changes in oral morphine equivalent daily dose over time. All linear mixed models used restricted maximum likelihood estimations and adopted an intention-to-treat approach. Among both cohorts, odds of readmission within 2 weeks based on exposure to intervention or control was examined using univariate logistic regression. Sensitivity analyses stratified patients by site (Box Hill Hospital or non-Box Hill Hospital sites), unit (surgical, general medicine, or rehabilitation), and psychological comorbidity (yes or no). An α of 0.05 was adopted for all analyses.

## 3. Results

### 3.1. Participants and descriptive data

**Figure 1.**
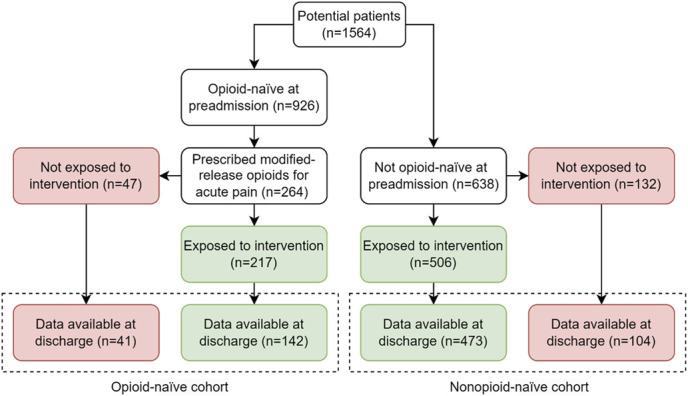
Study flowchart.

Among 1,564 potential patients, 183 and 577 patients were included in the opioid-naïve and nonopioid-naïve cohorts, respectively (Fig. 1). Reasons for exclusion were either that patients were not prescribed modified-release opioids (n = 662) or missing data (n = 142). Patient characteristics by cohort are summarized in Table 1. Among the total sample (n = 760), the mean (SD) age was 71.9 (19.1) years and hospital length of stay was 11.0 (14.6) days. Patients were mainly female (63%) and had no history of smoking (79%), alcohol use (84%), illicit drug use (96%), or psychological comorbidity (72%). When compared with the nonopioid-naïve cohort, the opioid-naïve cohort were younger in age, less likely to have a history of smoking, and less likely to have any psychological comorbidity (Table 1). There were no significant between-cohort differences for sex, alcohol use history, illicit substance use history, or hospital length of stay (Table 1). Patient characteristic by site and unit are shown in supplemental digital content (see Supplement B, http://links.lww.com/PR9/A348).

**Table 1:** Patient (n = 760) characteristics.

	Opioid-naïve cohort (n = 183)	Nonopioid-naïve cohort (n = 577)	*P*
Age, y	69.2 (20.1)	72.8 (18.7)	**0.030**
Female, n (%)	106 (57.9)	374 (64.9)	0.087
Smoking history, n (%)	25 (13.7)	133 (23.2)	**0.006**
Alcohol use history, n (%)	32 (17.6)	91 (15.8)	0.569
Illicit substance use history, n (%)	4 (2.2)	28 (4.9)	0.118
Any psychological comorbidity, n (%)	37 (20.2)	174 (30.4)	**0.007**
Hospital length of stay, d	9.6 (11.5)	11.4 (15.4)	0.145

Data are mean (SD), count (percentage within-group), or between-group *P*-value (independent *t* test or χ^2^ test). Bold: *P* < 0.05.

### 3.2. Outcome data

#### 3.2.1. Opioid-naïve cohort

Most (78%) were exposed to the intervention. Approximately 39% (n = 71) were discharged on a modified-release opioid. The mean (SD; range) oral morphine equivalent daily dose was 9.4 (14.4; 0–60) mg at discharge. Approximately 6.8% (n = 5/74) of patients met 2-week readmission criteria.

#### 3.2.2. Nonopioid-naïve cohort

Most (82%) were exposed to the intervention. Most (79%, n = 456) were discharged on a modified-release opioid. The mean (SD; range) oral morphine equivalent daily dose was 39.0 (67.3; 0–1280) mg at preadmission and 24.3 (28.9; 0–170) mg at discharge. Approximately 10% (n = 24/233) of patients met 2-week readmission criteria.

### 3.3. Main results

#### 3.3.1. Opioid-naïve cohort

The intervention led to 77% lower odds of discharge on a modified-release opioid compared with control (OR [95% CI]: 0.23 [0.11–0.49], *P* < 0.001). There were no significant between-group differences in readmission within 2 weeks (OR [95% CI]: 0.76 [0.08–7.45], *P* = 0.813).

#### 3.3.2. Nonopioid-naïve cohort

**Figure 2.**
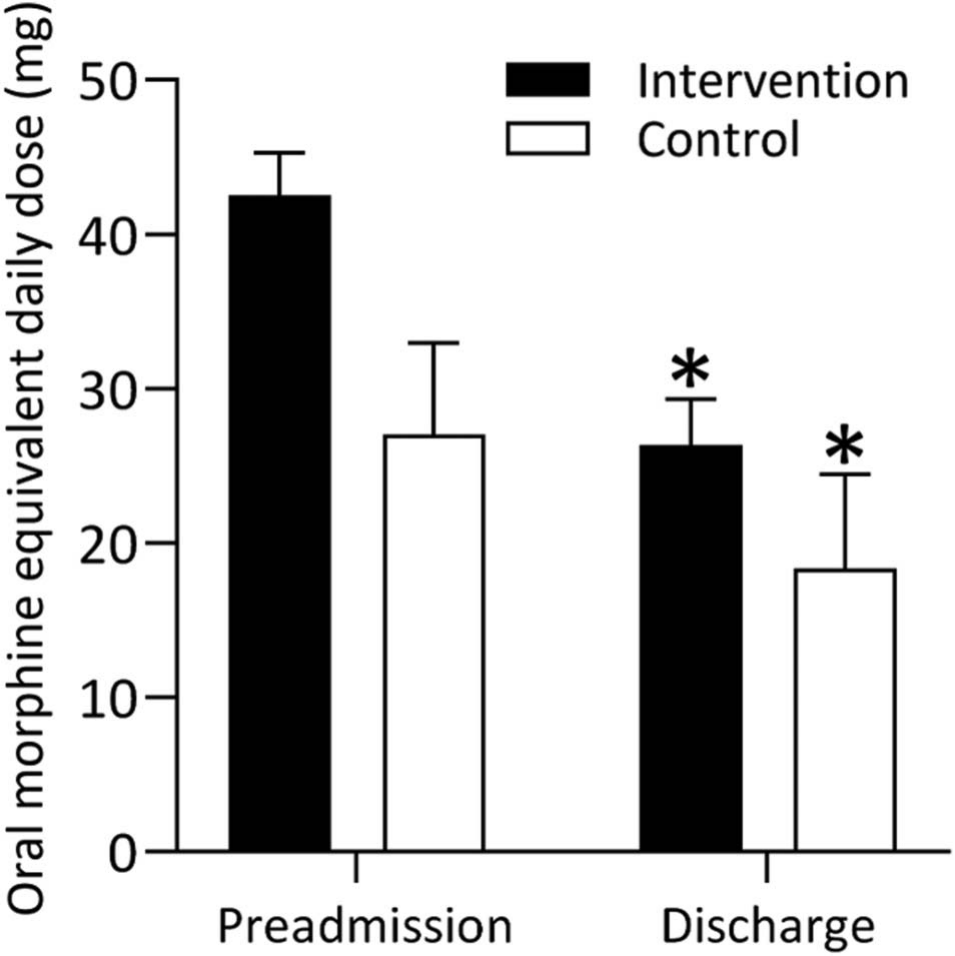
Change in oral morphine equivalent daily dose (mg) from preadmission to discharge among the nonopioid-naïve cohort exposed to the intervention or control. Data are estimated marginal mean and standard error from separate linear mixed models. *P < 0.05 compared with preadmission.

The intervention led to 61% lower odds of readmission within 2 weeks (OR [95% CI]: 0.39 [0.15–0.98], *P* = 0.045). There were no significant between-group differences in oral morphine equivalent daily dose from preadmission to discharge (estimated marginal mean net difference [95% CI]: −7.46 [−16.74 to 1.82] mg, *P* = 0.115). Both the intervention (estimated marginal mean net difference [95% CI]: −16.15 [−20.28 to −12.01] mg, *P* < 0.001) and control (estimated marginal mean net difference [95% CI]: −8.69 [−17.00 to −0.38] mg, *P* = 0.040) led to within-group reductions in oral morphine equivalent daily dose from preadmission to discharge (Fig. 2).

### 3.4. Other analyses

#### 3.4.1. Opioid-naïve cohort by site, unit, and psychological comorbidity

Sensitivity analyses for the opioid-naïve cohort by site, unit, and psychological comorbidity are summarized in Table 2. Between-group differences in discharge on modified-release opioid remained significant for patients at Box Hill Hospital, in general medicine units, in surgical units, and with no psychological comorbidity, yet not patients at non-Box Hill Hospital sites or with psychological comorbidity. Between-group differences in readmission within 2 weeks remained nonsignificant when patients were stratified by site, unit, or psychological comorbidity. Data paucity precluded sensitivity analyses for modified-release opioid at rehabilitation units, as well as readmission within 2 weeks at non-Box Hill Hospital sites, in general medicine units, in rehabilitation units, and with psychological comorbidity. Exploratory sensitivity analyses for the opioid-naïve cohort by specific surgical unit are shown in supplemental digital content (see Supplemental C, http://links.lww.com/PR9/A348).

**Table 2:** Sensitivity analyses for the opioid-naïve cohort by site and unit.

	Odds ratio (95% CI)	*P*
Box Hill Hospital (n = 137)		
Discharge on modified-release opioid	0.18 (0.08–0.43)	**<0.001**
Readmission within 2 weeks	0.69 (0.07–7.25)	0.755
Not Box Hill hospital (n = 46)		
Discharge on modified-release opioid	0.33 (0.06–1.78)	0.199
Readmission within 2 weeks	—	—
General medicine unit (n = 78)		
Discharge on modified-release opioid	0.18 (0.05–0.69)	**0.012**
Readmission within 2 weeks	—	—
Surgical unit (n = 95)		
Discharge on modified-release opioid	0.10 (0.03–0.30)	**<0.001**
Readmission within 2 weeks	0.57 (0.05–7.14)	0.664
Rehabilitation unit (n = 10)		
Discharge on modified-release opioid	—	—
Readmission within 2 weeks	—	—
Psychological comorbidity (n = 37)		
Discharge on modified-release opioid	0.25 (0.03–2.49)	0.237
Readmission within 2 weeks	—	—
No psychological comorbidity (n = 146)		
Discharge on modified-release opioid	0.19 (0.09–0.43)	**<0.001**
Readmission within 2 weeks	0.65 (0.06–6.94)	0.723

Data are odds ratio (95% CI) or between-group *P*-value (logistic regression). Bold: *P* < 0.05.

#### 3.4.2. Nonopioid-naïve cohort by site, unit, and psychological comorbidity

Sensitivity analyses for the nonopioid-naïve cohort by site, unit, and psychological comorbidity are summarized in Table 3. Between-group differences in oral morphine equivalent daily dose from preadmission to discharge remained nonsignificant when patients were stratified by site, unit, or psychological comorbidity. Sensitivity analyses revealed that between-group differences in readmission within 2 weeks remained significant among patients with psychological comorbidity, yet were no longer significant when patients were stratified by site, unit, or having no psychological comorbidity. Data paucity precluded sensitivity analyses for readmission within 2 weeks at rehabilitation units. Exploratory sensitivity analyses for the nonopioid-naïve cohort by specific surgical unit are shown in supplemental digital content (see Supplement D, http://links.lww.com/PR9/A348).

**Table 3:** Sensitivity analyses for the nonopioid-naïve cohort by site and unit.

	Effect estimate (95% CI)	*P*
Box Hill Hospital (n = 404)		
OMEDD from preadmission to discharge, mg	MD: −8.75 (−18.38 to 0.89)	0.075
Readmission within 2 weeks	OR: 0.38 (0.13 to 1.11)	0.076
Not Box Hill Hospital (n = 173)		
OMEDD from preadmission to discharge, mg	MD: −4.89 (−24.55 to 14.77)	0.626
Readmission within 2 weeks	OR: 0.33 (0.05 to 2.33)	0.268
General medicine unit (n = 333)		
OMEDD from preadmission to discharge, mg	MD: −9.57 (−21.62 to 2.48)	0.120
Readmission within 2 weeks	OR: 0.37 (0.09 to 1.56)	0.175
Surgical unit (n = 212)		
OMEDD from preadmission to discharge, mg	MD: −4.97 (−18.46 to 8.51)	0.470
Readmission within 2 weeks	OR: 0.79 (0.19 to 3.28)	0.748
Rehabilitation unit (n = 31)		
OMEDD from preadmission to discharge, mg	MD: 5.98 (−69.02 to 80.98)	0.876
Readmission within 2 weeks	—	—
Psychological comorbidity (n = 174)		
OMEDD from preadmission to discharge, mg	MD: −8.67 (−24.01 to 6.67)	0.268
Readmission within 2 weeks	OR: 0.14 (0.03 to 0.69)	**0.015**
No psychological comorbidity (n = 398)		
OMEDD from preadmission to discharge, mg	MD: −5.78 (−16.49 to 4.93)	0.290
Readmission within 2 weeks	OR: 0.59 (0.17 to 2.03)	0.406

Data are odds ratio (OR; 95% CI), estimated marginal mean net difference (MD; 95% CI), or between-group *P*-value (logistic regression or linear mixed model). Bold: *P* < 0.05.

OMEDD, oral morphine equivalent daily dose.

## 4. Discussion

This study showed exposure to the intervention (organisation- and patient-level analgesic stewardship program interventions) led to improvements in discharge on a modified-release opioid, yet not readmission within 2 weeks, among the opioid-naïve cohort when compared with control (organisation-level analgesic stewardship program interventions alone). Among the nonopioid naïve cohort, the intervention led to improvements in readmission within 2 weeks, yet not oral morphine equivalent daily dose from preadmission to discharge. Sensitivity analyses revealed that site (Box Hill Hospital or non-Box Hill Hospital sites), unit (surgical, general medicine, or rehabilitation), and psychological comorbidity were associated with effectiveness.

Our study showed that the addition of patient-level analgesic stewardship program interventions led to lower odds of discharge on a modified-release opioid among the opioid-naïve cohort and lower odds of readmission within 2 weeks among the nonopioid-naïve cohort. While we are unaware of any studies that also specifically examined the benefits of adding patient-level interventions, our observations align with conclusions from a systematic review of 43 studies that analgesic stewardship programs can improve opioid prescribing at discharge. ^19^ However, our findings challenge the notion that multifaceted strategies are unlikely to yield effects greater than that of simpler approaches such as the implementation of guidelines. ^19^ Notably, the reduced odds of discharge on a modified-release opioid in our study were approximately 58% greater than the previously reported effects of implementing an organisational position statement on modified-release opioid prescribing within 2 Australian hospitals. ^6^ Given the potential greater comparative effectiveness, quantifying the cost-effectiveness of adding patient-level interventions is recommended for the design of future analgesic stewardship programs.

Determining clinical meaningfulness of analgesic stewardship programs remains contentious, and the most recent recommendations from the Initiative on Methods, Measurement, and Pain Assessment in Clinical Trials ^12^ support monitoring opioid-related adverse outcomes rather than opioid consumption. Hence, our observation that the addition of patient-level interventions led to lower odds of readmission within 2 weeks in nonopioid naïve patients may be clinically meaningful as reducing avoidable hospital readmissions is a common priority of healthcare systems such as Australia. ^4^ Future evaluations of analgesic stewardship programs could benefit from collecting data on a range of opioid-related adverse outcomes, such as nausea or opioid use disorder, that are identified as important to consumers (eg, patients and caregivers) and end-users (eg, doctors, nurses, and pharmacists).

Our sensitivity analyses suggested that site, unit, and psychological comorbidity may be effect modifiers. This may in part be explained by the geographical location of each hospital, which per most recent Australian estimates can account for approximately a 5-fold magnitude of variation in opioid dispensing. ^5^ Another explanation is the way in which the analgesic stewardship program was progressively implemented at our health service. For example, while Box Hill Hospital commenced in August 2022, the final site (Peter James Centre) did not commence until February 2023, which may indicate that effectiveness accrues over time as programs become more entrenched. Furthermore, the proportion of patients from each unit differed by hospital (*P* < 0.001; general medicine: 33%–68%; surgical: 26%–63%; rehabilitation 0%–100%). The proportion of patients with any psychological comorbidity was also more than 2-fold greater (*P* = 0.004) at non-Box Hill Hospital sites (see Supplement B, supplemental digital content, http://links.lww.com/PR9/A348). As for psychological comorbidity, our observations may in part stem from the increased likelihood of opioid prescription among adults with mental ill-health. ^8^ However, these findings should be interpreted with caution, given we excluded patients who received clinical input/recommendation on analgesia from a specialist treating team or were prescribed methadone, both of which are associated with psychological comorbidity. ^16^ Collectively, these data suggest that site-, unit-, and population-specific strategies may warrant consideration when designing and implementing analgesic stewardship programs.

A key strength of our study was the inclusion of parallel control groups given the omission in most (84%, n = 36/43) previous studies identified in a systematic review. ^19^ Moreover, the inclusion of patients from multiple sites (hospitals) and units (general medicine, surgery, or rehabilitation) markedly heightened ecological validity. Although, our study had several limitations. First, we lacked a true control, and therefore, our estimates of comparative effectiveness were potentially of smaller magnitude than the true effect of the intervention. By contrast, the lack of randomisation and potential selection bias whereby higher-risk patients were potentially more likely to receive patient-level interventions may have increased the magnitude of the observed effect. Second, we only examined sites at one health service and given our sensitivity analyses suggested potential effect modification, generalisation to other hospitals and health services may be limited. Extrapolation internationally should also be performed with caution given global heterogeneity in opioid use. ^15^ Finally, examination of cost-effectiveness and sustainability was beyond the scope of our study and warrants attention and comparison with simpler interventions.

A multidisciplinary analgesic stewardship program comprising both organisation- and patient-level interventions may lead to better opioid prescribing at discharge and potentially clinically meaningful reductions in readmission when compared with organisation-level interventions alone. Opioid-naïve status at preadmission, as well as site, unit of admission, and the presence of psychological comorbidity may be effect modifiers. Further analysis of cost-effectiveness and sustainability of analgesic stewardship programs, especially among patients under the care of specialist pain teams, is warranted.

## Disclosures

The authors have no conflict of interest to declare.

## Supplemental digital content

Supplemental digital content associated with this article can be found online at http://links.lww.com/PR9/A348.
